# Metaplastic squamous cell breast cancer: A case report and treatment strategy during covid-19 pandemic

**DOI:** 10.1016/j.ijscr.2021.01.079

**Published:** 2021-01-22

**Authors:** G. Tomasicchio, A. Rizzi, L.S. Stucci, M. Moschetta, G. Giliberti, C. Punzo

**Affiliations:** a Division of Surgery, Department of Emergency and Organ Transplant (DETO), University “Aldo Moro” Medical School of Bari, Bari, Italy; b Medical Oncology Unit, Policlinico Hospital, Bari, Italy; c Division of Diagnostic Imaging, Department of Emergency and Organ Transplantation (DETO), Aldo Moro University, Medical School, Bari, Italy; d Division of Pathology, Department of Emergency and Organ Transplantation (DETO), Aldo Moro University, Medical School, Bari, Italy

**Keywords:** UIQ, upper inner quadrant, UOQ, upper outer quadrant, Breast cancer, Covid-19, Multidisciplinary management, Squamosus carcinoma

## Abstract

**Introduction:**

Squamous carcinoma is a very rare breast cancer resulting from a differentiated squamous metaplasia. Its diagnosis is late due to the absence of specific clinical and radiological features.

**Presentation of case:**

The authors present the case of a 39-year-old patient with the clinical onset of an inflammatory cyst diagnosed with mammography and cytological examination. After segmentectomy, on histological examination was defined as: “squamous keratinizing aspect”. In light of this report she was subjected to quadrantectomy with lymphadenectomy. She did not undergo radiotherapy treatment, which was delayed due to the ongoing pandemic. After a few months developed a local recurrence and underwent a mastectomy with multidisciplinary treatment.

**Discussion:**

Squamously-differentiated metaplastic carcinoma of the breast is one of the rarest forms of breast cancer. Its etiopathogenesis is still unclear but some authors suggest that it originates from a squamous metaplasia deriving from the epithelium of cysts. This is a very aggressive and fast-growing tumour especially in young women, in fact the patient had a recurrence after a few months. She came to our attention 6 months after the initial surgery with a relapse at a local-regional level for which she was subjected to radical treatment and with a metastatic spread for which she was given the first-line chemotherapy treatment.

**Conclusion:**

There are no well-defined guidelines for the treatment of this rare form of cancer. The case illustrates how the rapid aggressiveness of the disease requires adequate and immediate multidisciplinary treatment from the very beginning.

## Highlights

• Squamous cell cancer is one of the rarest forms of breast cancer with an incidence of 0.1–0.4% of all breast malignancies.
• Clinical and imaging ﬁndings in squamous cell cancer can mimic those of a benign disease.
• Aggressive and rapidly-evolving tumour without specific clinical and radiological features.
• No well-defined guidelines for the treatment of this rare cancer form.
• Role of Covid-19 pandemic in the delay of correct oncological treatment.

## 1 Introduction

Squamous carcinoma of the breast is a rare neoplasm with an incidence of between 0.1% and 0.4% [1,2]. It is believed to develop from the squamous metaplasia of carcinomal ductal cells [2,3]. It has no specific clinical and radiological features that allow for early diagnosis. In the literature, since 1908, it is described as an aggressive and rapidly-evolving tumour, hormone receptor-negative and refractory to treatment with a poor prognosis. The aim of this report is to describe the clinical presentation of an aggressive squamous carcinoma as benign disease and discuss diagnosis and management, highlighting the effect of pandemic on oncological treatment. This case report was written according to SCARE guidelines [4].

## 2 Presentation of case

The patient was a 39-year-old woman, negative for neoplastic familiarity, nulliparous, without co-morbidities, smoker, with an allergic diathesis to antibiotics. She had no past history of breast pathology but she had never screened.

Via self-examination she discovered a new lump in the UIQ of her left breast. The mammography, performed elsewhere, showed an inflamed, 3 cm cystic formation on which a cytological examination was carried out, with a finding of “some neutrophilic granulocytes, numerous macrophages, stromal frustules and anucleated horny scales, a compatible cytological finding with material from cysts”.

In January 2020 she underwent a segmentectomy of the neoformation at another hospital, which, on histological examination was defined as a “sebaceous carcinoma with squamous keratinizing aspects, infiltrating the chorion, with undamaged margins”. In light of this report, she was subjected to a UOQ quadrantectomy of the left breast associated with lymphadenectomy. The definitive histological examination highlighted “inflammatory-granulomatous foci of a foreign body type, of a liponecrosis and lipogranulomatosis type, not showing any residual neoplasia. Lymph nodes free from neoplasia”. Subsequently, due to the health emergency due to the Covid-19 pandemic, the patient was unable to undergo radiotherapy treatment.

**Fig. 1:**
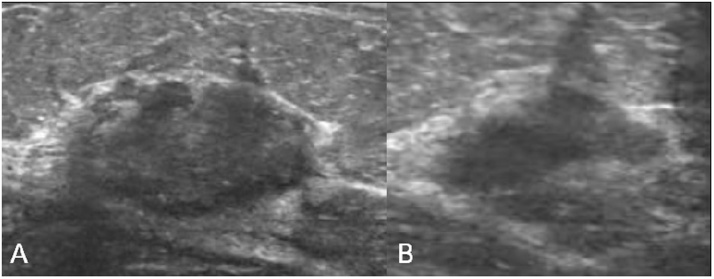
Ultrasound transverse (A) and sagittal (B) scans showing an hypoechoic inhomogeneous nodular lesion corresponding to the breast lump on the surgical scar at the subareolar region of the left breast.

In May, as a result of self-examination of peri-cicatricial nodules, she underwent a ultrasound check, at our Breast Unit, with the finding of an “oval formation of 20 × 13 mm, further solid contiguous formations. one caudal of 10 × 10 mm and the other cranial of 7 × 5 mm. There was an absence of adenopathies in the axillary area on both sides.” (Fig. 1)

She underwent a micro-histological examination via needle biopsy on the peri-cicatricial swellings with the finding of a “poorly differentiated infiltrating carcinoma of a non-special type, with a positive immunophenotype for EMA, GATA-3, p40, p63 and negative for cytokeratin 7 and GCDFP-15. There was an absence of immunoreactivity for oestrogen and progesterone receptors, with a Ki 67 equal to 50% and absence of membrane immunoreactivity for Her2/neu (clone CB11)”. Tumour markers showed a slightly increased value of 9.5 ng/mL for CEA, while the CA 15.3, CA 125 and Ca 19.9 were all normal.

**Fig. 2:**
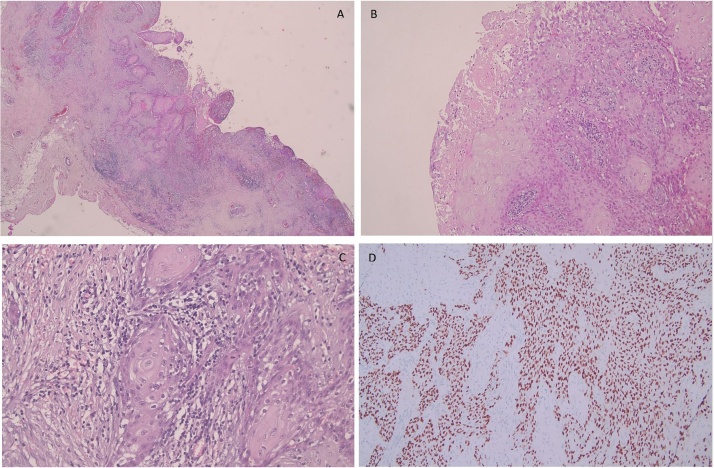
A) hematoxylin eosin staining; 40× magnification. B) hematoxylin eosin staining; 200× magnification. C) hematoxylin eosin staining; 400× magnification. D) p 63 marker. Immunohistochemistry; 200× magnification.

In July, at our operative unit the patient underwent a mastectomy with the placement of an expander with a confirmation of the definitive histological examination of an “infiltrating metaplastic cystic squamous carcinoma, with focal areas of sebaceous differentiation. Obvious aspects of peritumoural endovasal neoplastic permeation. CK 34Beta E 12 (+++), p63 (+++), CK 5/6 (+++), EMA (+++), S100 (+++), Oestrogen R (negative) Progesterone R (negative), Erb2neu (negative), GCDFP15 (negative), Ki67: 45%”. (Fig. 2)

At the post-operative CT check, lymph nodes with a maximum diameter of 8 mm in the left internal mammary chain and lymph nodes of 5 mm in the right mammary chain with some lymph nodes around a centimetre in diameter were found in the right axillary site. In consideration of the prognostic risk and the clinical evolution of the disease, it was decided to perform an in-depth PET CT with evidence of “areas of radiopharmaceutical hyperaccumulation the greatest in intensity in the subcarinal area (SUV max 3.0) and in the Barety space (SUV max 2.9), the others in correspondence to the left internal mammary chain (SUV max 2.2) and in the retrosternal area (SUV max 2.8), compatible in the first hypothesis with adenopathies of secondary significance.”

The patient was judged to be in a metastatic phase in the lymph nodes and on the basis of this clinical picture, the carrying out of an immunohistochemical expression analysis on a tumour sample of the PDL-1 marker was required to assess the patient's eligibility for first-line therapy according to the “Atezolizumab + Nab-Paclitaxel” protocol, in case of PDL-1 positivity> 1% [5]; This analysis was negative. She also underwent a search for the genetic mutation for Brca1/2 which proved negative.

On the basis of this clinical picture, the patient undertook first-line therapy following the "Nab-Paclitaxel" protocol [6].

She is currently under strict follow-up at Oncology Unit of our Breast Unit, without disease progression.

## 3 Discussion

Metaplastic squamous carcinoma of the breast is one of the rarest forms of breast cancer. Its etiopathogenesis is still unclear but some authors suggest that it originates from a squamous metaplasia deriving from the epithelium of cysts or chronic abscesses. Other authors, however, hypothesize that it may originate from myoepithelial cells [7,8]. In our experience, we observed another case of squamous carcinoma in an elderly woman, in whom the clinical onset was associated with an inflammatory cyst [9].

It has not yet proved possible to identify mammographic or ultrasound features that allow an early diagnosis but it has been found that in more than 50% of cases it tends to present as a cystic formation. [1,10]. The pre-operative diagnosis can only be performed by means of a micro-histological examination on needle biopsy [1,2], although only the definitive histological examination can really characterize a state of pure squamous. Other authors have also shown that it can present larger than other forms of breast cancer, up to 8 cm [1,7,10,11]. Squamous carcinoma mainly affects postmenopausal women but there are also rare cases in younger women (<45 years old) [2,7,12]. This is a very aggressive and fast-growing tumour especially in young women, patient had a recurrence after a few months. In only 10–30% of cases are the axillary lymph nodes positive. The absence of lymph node metastases is considered a positive prognostic factor, although in more than 30% of cases the patients develop secondary localizations due to the presence of endovasal neoplastic permeation that favours its spread via the bloodstream [1,7,11]. Positivity for cytokeratin 5/6 and for CK 34 beta E12 indicate the squamous origin of the neoplasm [2]. In most cases the tumour is negative for oestrogen and progesterone receptors without Her2/neu overexpression, as found in our patient [2,10].

There are no well-defined guidelines for the treatment of this rare form of cancer. Some authors have shown that it is resistant to common chemotherapeutic agents and that there are no differences between patients who have undergone neoadjuvant or adjuvant therapy compared to those who have not received it [13]. At the same time, other authors have demonstrated the efficacy, albeit limited, of adjuvant therapy by focusing on drugs such as TS-1, CDDP, eribulin and platinum-based agents in the treatment of this pathology. [1,11,14]. In the case reported, the role of radiotherapy remains uncertain, which if it had been performed in adequate time and not delayed by the Covid-19 pandemic, might perhaps have prevented the onset of a relapse.

The likelihood of developing distant metastases is not a rare event and treatment with chemotherapy often does not induce advantages in terms of OS and PFS SCC. Therefore, the molecular study of these rare forms may be useful in the search for currently unknown mutations that can potentially treated by target therapy [15].

Most authors agree on the need for primary surgical therapy over others. The rarity and characteristics of this neoplasm require personalized treatments, widely discussed by a multidisciplinary team such as those in the Breast Units. In the case of squamous carcinoma, in the elderly patient previously treated by us, the surgical therapy was followed by adjuvant chemo- and radiotherapy and after a close follow-up of 56 months the patient is still disease-free. In the case described here, the 39-year-old patient came to our attention 6 months after the initial surgery with a relapse at a local-regional level for which she was subjected to radical treatment and with a metastatic spread for which she was given the first-line chemotherapy treatment.

## 4 Conclusion

This case provides the perception of the rapid progression of metaplastic squamous cell tumours and the need for these particular forms to be recognized and diagnosed in adequate time. Primary treatment should include demolitive surgery and be treated in specialized multidisciplinary facilities. Unfortunately, the period of the pandemic led the patient under examination to delay treatment and not fully understand the aggressiveness of the disease.

## Declaration of Competing Interest

All authors negate any conflict of interest

## Funding

This research did not receive any specific grant from funding agencies in the public, commercial, or not-for-profit sectors.

## Ethical approval

The study is exempt from ethnical approval in our institution.

## Consent

Written informed consent was obtained from the patient for publication of this case report and accompanying images. A copy of the written consent is available for review by the Editor-in-Chief of this journal on request

## Author contribution

C. Punzo: Senior advisor, supervision, performed surgery.

G. Tomasicchio: data analysis, study concept and design, writing the paper.

A. Rizzi: Study concept and design.

M. Moschetta: Data collection, data analysis.

L. S. Stucci : Data collection, data analysis

G. Giliberti : Data collection, data analysis

## Registration of research studies

Not applicable.

## Guarantor

Professor Punzo Clelia.

## Provenance and peer review

Not commissioned, externally peer-reviewed.
